# Minimised SARS-CoV-2 transmission during Tokyo 2020 Olympic and Paralympic Games: insights from enhanced integrated surveillance, Japan, July to September 2021

**DOI:** 10.2807/1560-7917.ES.2026.31.42.2600009

**Published:** 2026-10-22

**Authors:** Munehisa Fukusumi, Keiko Tsukada, Kanako Otani, Masato Yamauchi, Yoshiyuki Sugishita, Chiaki Ikenoue, Tsuyoshi Sekizuka, Tomoe Shimada, Taro Kamigaki, Motoi Suzuki, Tomimasa Sunagawa, Makoto Kuroda, Tomoya Saito

**Affiliations:** 1 Center for Public Health Action in Applied Epidemiology, National Institute of Infectious Diseases, Japan Institute for Health Security, Tokyo, Japan; 2 Center for Emergency Preparedness and Response, National Institute of Infectious Diseases, Japan Institute for Health Security, Tokyo, Japan; 3 Department of Infectious Disease Surveillance, National Institute of Infectious Diseases, Japan Institute for Health Security, Tokyo, Japan; 4 Project for Elimination of Lymphatic Filariasis Phase 2, Japan International Cooperation Agency, Tokyo, Japan; 5 Medical Policy Division, Bureau of Public Health, Tokyo Metropolitan Government, Tokyo, Japan; 6 SEKIZUKA Industry Co., Ltd., Tokyo, Japan; 7 Center for Infectious Disease Epidemiology, National Institute of Infectious Diseases, Japan Institute for Health Security, Tokyo, Japan; 8 Department of Medical Technology, Faculty of Health Sciences, Kumamoto Health Science University, Kumamoto, Japan

**Keywords:** COVID-19 pandemic, international sporting event, mass gathering, whole- genome sequencing, enhanced surveillance, PCR test

## Abstract

**BACKGROUND:**

Amid the COVID-19 pandemic, stringent public health measures were taken during the Tokyo 2020 Olympic and Paralympic Games (Tokyo 2020).

**AIM:**

We describe SARS-CoV-2 transmission among Games participants and in-person contributors (GPIPC)s.

**METHODS:**

Tokyo 2020-related SARS-CoV-2 infection cases were individuals linked to the event, testing positive for the virus between 1 July−19 September 2021, reported to the Tokyo Metropolitan Government. Attack rates among athletes and team officials were determined. Enhanced case-based surveillance integrating genomic sequencing data supported viral spread investigations among various GPIPC groups.

**RESULTS:**

A total 804 cases (median age: 35 years, interquartile range (IQR): 27–45; 194 female/610 male) were reported. Among athletes and team officials, attack rates were 0.6% (65/11,400) in Olympic Games and 0.6% (30/4,800) in Paralympic Games. These 95 infections concerned mainly overseas travellers (n = 92), 85 of whom were diagnosed within 14 days of arrival in Japan. Among the 804 cases, whole-genome sequencing data were obtained for 304 (median age: 38 years, IQR: 29–47; 67 female/237 male) with sufficient viral load (as indicated by RT-PCR Cq ≤ 32), including 79% (75/95) of athletes and team officials cases, 74% (131/176) of Games’ organisers and media cases, and ˂ 20% (98/533) of remaining cases. Delta variants dominated (94%; 285/304), with domestic (AY.29 lineage) Delta strains prominent among Delta-variant-affected resident cases (96%; 104/108) and Games’ organisers and media cases diagnosed ≥ 15 days after entering Japan (97%; 63/65). Overseas Delta strains mostly affected travellers within 14 days of entry, particularly athletes and team officials (97%; 66/68) and Games’ organisers and media (29/34).

**CONCLUSION:**

Multilayered-risk-reduction measures likely minimised SARS-CoV-2 transmission to athletes.

**Key public health message**

**What did you want to address in this study?**

Amid the COVID-19 pandemic, the strict public health measures of the Tokyo 2020 Olympic and Paralympic Games led Games participants and in-person contributors to be in a cohort-like environment. This created an opportunity to retrospectively assess SARS-CoV-2 transmission among these individuals, particularly athletes and team officials, using epidemiological and genomic surveillance data.

**What have we learnt from this study?**

Among athletes and team officials 0.6% (65/11,400) tested SARS-CoV-2 positive in the Olympic Games and 0.6% (30/4,800) in the Paralympic Games. These 95 infections mainly concerned travellers from abroad (i.e. 85) diagnosed at arrival or within 14 days of entry in Japan. Of these travellers, 68 were affected by Delta variants, including 66 with an overseas Delta strain and two with a domestic strain.

**What are the implications of your findings for public health?**

Among athletes and team officials, low rates of infections, mainly affecting those from abroad shortly after their arrival in Japan indicate that the Games’ unique infection-control measures effectively minimised SARS-CoV-2 transmission. Few domestic viral strains were moreover detected in this group. Integrated epidemiological and genomic surveillance allows assessing mitigation strategies in international mass gatherings.

## Introduction

Under the global pandemic of COVID-19, the Tokyo 2020 Olympic and Paralympic Games (Tokyo 2020) were held from 23 July to 8 August and from 24 August to 5 September 2021, respectively, as the largest international sporting event conducted during Japan’s most substantial COVID-19 surge [1]. There were 43 venues located mostly in the Tokyo Metropolitan Area, with additional sites in eight prefectures. The Tokyo 2020 Games were held without spectators and the number of Games-related travels to Japan from overseas was substantially reduced as part of the implemented risk mitigation measures. As a result, approximately 33,000 individuals travelled from overseas, related to the Olympics, including, among others, around 11,400 athletes and team officials, 3,600 Games’ organisers, and 14,200 media personnel. In addition, some 10,000 individuals linked with the Paralympics, included notably 4,800 athletes and team officials, 1,100 Games’ organisers, and 2,700 media personnel [2]. All 47 prefectures in Japan accepted Games’ delegations as host towns before the Games.

Under the ongoing COVID-19 pandemic, Japan enhanced COVID-19 surveillance beyond the existing system and expanded capacity for whole-genome sequencing (WGS) of SARS-CoV-2 [3]. Combined countermeasures were also implemented to prevent SARS-CoV-2 transmission among Games participants and in-person contributor (GPIPCs), and to mitigate its impact on domestic residents and vice−versa, including a 14-day quarantine upon entry into Japan, strict management of athletes to minimise contact with non-athlete groups during all activities, regular screening tests from arrival to departure for active case finding and early case isolation, and infection-prevention measures at venues; e.g. wearing masks and physical distancing. Additional measures included ensuring compliance with the rules by creating a ‘Playbook’ [4] and establishing operational structures for the Games and a rapid response system in the event of a COVID-19 outbreak, in collaboration with relevant GPIPCs involved [5].

The unique conditions of Tokyo 2020, in which GPIPCs were in a cohort-like situation due to stringent public health measures, presented a suitable opportunity to elucidate the transmission dynamics of SARS-CoV-2 in this large-scale international mass gathering through genomic surveillance. Thus, the aim of the study was to investigate SARS-CoV-2 transmission dynamics, combining epidemiological data with comparative genomic findings in Tokyo 2020 during the COVID-19 pandemic.

## Methods

### Study design

This study was a retrospective, descriptive analysis of data collected through integrated enhanced national case-based surveillance, genomic surveillance and epidemiological investigations conducted under the jurisdiction of the Tokyo Metropolitan Government (TMG).

### Case definition and categorisation of Games participants and in-person contributors

Cases of SARS-CoV-2 infection related to Tokyo 2020 were defined as those who tested positive for SARS-CoV-2 by PCR (following positive or inconclusive saliva antigen screening), between 1 July and 19 September 2021 (data were retrieved on 4 October 2021) and were reported to the Health Center Real-time Information-sharing System on COVID-19 (HER-SYS) under the jurisdiction of TMG. 

The GPIPCs were categorised into four groups: ‘athletes and team officials’, ‘Games’ organisers and media’, ‘other GPIPCs’ and ‘unclassifiable personnel’, based on their roles and characteristics as defined by risk categories in the Tokyo 2020 Playbook [4] (Table 1).

**Table 1:** Games participants and in-person contributors categories related to the Tokyo 2020 Olympic and Paralympic Games, Japan, 1 July−19 September 2021 (n = 4 categories)

Category	Attributes
Athletes and team officials	Athletes and national team staff, including team doctors, coaches, trainers and caregivers for para-athletes
Games’ organisers and media	International Olympic Committee officials, International Paralympic Committee officials, International Sports Federation officials, referees, broadcasting services and press operations
Other GPIPCs	GPIPCs not classified into the above two categories, including TOCOG staff, volunteers, contractors, interpreters and torch relay participants
Unclassifiable personnel	Those who could not be categorised into the above three categories due to incomplete or unclear information for proper classification

GPIPCs: Games participants and in-person contributor; TOCOG: Tokyo Organizing Committee of the Olympic and Paralympic Games.

### Surveillance and screening systems for the Tokyo 2020 Games

Under the ongoing COVID-19 pandemic, Japan extended COVID-19 surveillance beyond the existing system, with increased capacity for whole-genome sequencing (WGS) of SARS-CoV-2 (Table 2) [3].

**Table 2:** Surveillance and screening systems for COVID-19 for the Tokyo 2020 Olympic and Paralympic Games, Japan, 1 July−19 September 2021

System	System description
Enhanced national case-based surveillance	From 1 July to 19 September 2021, laboratory-confirmed SARS-CoV-2 infections were required to be registered in the national-level HER-SYS by medical doctors who diagnosed the case or by public health centres after receiving reports from physicians via electronic or paper-based reporting forms under the Infectious Diseases Control Act [6]. Case data included demographic, clinical, and laboratory information; and exposure history. From 1 July until 19 September 2021, in addition to routine procedures, in response to a special request by the MHLW for SARS-CoV-2 infections related to Tokyo 2020, public health centres were required to indicate cases in the newly created ‘Olympic and Paralympic Games’ check box; the so-called Tokyo 2020 flag in HER-SYS [3].
National genomic surveillance	According to administrative notifications [20], specimens from SARS-CoV-2 infections among Tokyo 2020 related GPIPCs were collected in accordance with the Infectious Diseases Control Act and WGS was conducted in collaboration between a commercial laboratory and the Pathogen Genomics Center of the NIID. Specimens collected at the Polyclinic in the Athletes' Village during Tokyo 2020 were sent to a commercial laboratory contracted by the TOCOG, where WGS was performed. The WGS results were subsequently sent to the Pathogen Genomics Center at NIID for further analysis. Specimens collected at sites other than the polyclinics, including quarantine stations, were sent directly to NIID for sequencing.
Pre-entry and entry-screening test at quarantine stations	The Japanese government mandated that all overseas GPIPCs provide proof of two negative SARS-CoV-2 test results within 96 hours before entry into Japan, with one test conducted within 72 hours of departure. Additionally, a PCR entry-screening test was performed upon arrival in Japan [5]. The SARS-CoV-2 infections confirmed by PCR testing were reported to HER-SYS.
Large-scale screening for GPIPCs designated to TOCOG	Large-scale screening tests for SARS-CoV-2 were conducted by TOCOG for all designated GPIPCs. Daily screening testing procedures were specially tailored for GPIPCs [21]. The frequency of screening tests depended on the risk categories in Tokyo 2020 [4], determined considering both the risk of them infecting others and their necessity to the operation of the Games. Confirmed SARS-CoV-2 infections by PCR testing were reported to HER-SYS.

GPIPCs: Games participants and in-person contributors; HER-SYS: Health Center Real-time Information-sharing System on COVID-19; MHLW: Ministry of Health, Labour and Welfare; NIID: National Institute of Infectious Diseases; TOCOG: Tokyo Organizing Committee of the Olympic and Paralympic Games; Tokyo 2020: Tokyo 2020 Olympic and Paralympic Games; WGS: whole-genome sequencing.

### Monitoring of Games participants and in-person contributors and infection control measures

#### Entry quarantine procedures

During the Tokyo 2020, overseas GPIPCs were required to be tested for SARS-CoV-2 pre-departure to Japan (Table 2). They also underwent testing on arrival, and health screening. After entry, they were subject to a 14-day period of enhanced monitoring with movement restriction, regular testing, and daily health reporting in the Athletes’ Village or designated accommodation. Notably, athletes were managed under a separate operational track from others to minimise contact. Athletes and team officials who tested positive were quarantined individually, as were their close contacts for 14 days after their last contact.

#### Screening test for COVID-19 for Games participants and in-person contributors

At the Tokyo 2020 COVID-19 screening was implemented using a risk-based approach according to GPIPCs’ role and degree of contact with athletes (Table 2). Athletes and team officials underwent daily testing, while other GPIPCs, including Games’ organisers, media personnel, staff, and volunteers, were tested daily, every 4 days, or every 7 days depending on their operational role and proximity to athletes. Cases were confirmed through quantitative saliva antigen screening, with PCR confirmation for positive or inconclusive results.

#### Infection prevention and control measures at the Tokyo 2020

Athletes and team officials were subject to enhanced measures. They stayed in the Athletes’ Village or other designated accommodation and followed structured daily routines, including designated locations and times for training. Dedicated areas, such as athlete dining facilities, were modified with droplet prevention barriers, and food service was managed by staff to reduce contact. Physical distancing was enforced in operational areas, such as mixed zones where different categories of GPIPCs could be present (e.g. when athletes interacted with media and broadcast personnel, primarily for interviews after competition). Moreover, spaces used by athletes were regularly disinfected and ventilated. These measures collectively constituted a so-called ‘bubble system’. In addition, confirmed SARS-CoV-2 infection cases were isolated individually in designated facilities, separated from their respective national delegations, and close contacts of confirmed COVID-19 cases were managed separately from other athletes, with restricted movement, dedicated staff accompaniment, and access to separate training facilities, allowing continued participation in competitions under controlled conditions. Games’ organisers and media personnel were required to limit their movements to pre-approved locations (e.g. competition venues, accommodation, and designated transport), avoid contact with the general public, and comply with regular testing and daily health reporting. Rather than routine consultation with a healthcare worker, daily health reporting was primarily based on self-monitoring of body temperature and other COVID-19 symptoms and communicating results through the Online Check-in and Health Reporting App (OCHA). In addition, body temperature was checked when entering Games venues; those with a temperature of 37.5°C or higher underwent a second check and, if the elevated temperature was confirmed, were not permitted to enter the venue and were referred for further assessment. Access to operational areas, including mixed zones, was restricted and physical distancing was required. Other GPIPCs, including staff and volunteers, were encouraged to adhere to standard infection prevention measures such as temperature monitoring, hand hygiene, surgical mask use, and minimising conversation during meals. Compliance with infection prevention measures was guided by a code of conduct. Although adherence relied largely on individual responsibility supported by on-site reminders, compliance among GPIPCs was enforced under the Playbook, with violations potentially resulting in penalties, including disqualification from the Games.

### Data collection

Data on cases related to Tokyo 2020 under the jurisdiction of TMG were extracted from HER-SYS [6] and integrated with information from multiple surveillance systems (Table 2) and epidemiological investigations. Data were shared between TMG and the National Institute of Infectious Diseases (NIID) under the cooperative framework for infection prevention and control, as well as public health response [7]. A comprehensive line list was created, incorporating variables such as demographic and clinical information, exposure histories from epidemiological investigations, and laboratory data, including PCR test results and genomic sequencing data.

### Genomic analysis

Whole-genome sequencing of SARS-CoV-2 was performed on samples with quantification cycle (Cq) values ≤ 32 using the PrimalSeq (ARTIC) multiplex reverse transcription PCR (RT-PCR) approach, followed by Illumina sequencing on the NextSeq 500 platform. Comparative genomic analysis was performed according to previously reported methods [8,9]. A maximum-likelihood (ML) phylogenetic analysis based on single-nucleotide (nt) variants (SNVs) was performed using iqtree v2.4.0 [10] with an ultrafast bootstrap test parameter, followed by visualisation using FigTree v1.4.4 [11].

### Integrated epidemiological and genomic analysis

We conducted an integrated epidemiological and molecular analysis of SARS-CoV-2 infections related to Tokyo 2020. The variables used in the analysis were sex (male/female) and age, date of symptom onset, date of diagnosis, residence status (overseas traveller or domestic resident), date of entry into Japan for those residing abroad, categories of GPIPCs related to Tokyo 2020 based on role and affiliation, symptoms, exposure history, clinical outcome at the time of diagnosis, RT-PCR test results and genomic sequencing results. Attack rates were calculated separately for the Olympic and Paralympic Games, with the respective number of participants used as denominators [2]. We used Excel and R 4.2.0 [12] to conduct analysis. Characteristics, WGS status, and the proportion of SARS-CoV-2 lineages in reported SARS-CoV-2 infections related to Tokyo 2020 were stratified by combinations of case category and residence status, to evaluate the effectiveness of infection prevention and control measures by comparing groups subject to different control strategies.

#### Cluster identifications

A ‘cluster’ was defined as an accumulation of five or more cases infected with SARS-CoV-2 with similar or identical genome sequences, differing by no more than two nt mutations, that were epidemiologically linked based on information obtained from investigations conducted by public health centres and the Tokyo Organizing Committee of the Olympic and Paralympic Games (TOCOG). The threshold of five or more cases was adopted based on previous studies in Japan [13]. Clusters were first identified based on genetic similarity and subsequently assessed for epidemiological links as ascertained from the comprehensive line list.

## Results

### Descriptive epidemiology

There were 804 cases reported (median age: 35 years; IQR: 27–45) with 24% and 76% being of female and male sex respectively. The cases included 232 (29%) overseas travellers and 572 (71%) domestic residents. No fatalities were reported at the time of diagnosis. The cases comprised 95 (12%) athletes and team officials, 176 (22%) Games’ organisers and media, 506 (63%) other GPIPCs and 27 (3%) unclassifiable personnel. Among athletes and team officials, for whom denominator data were available, the attack rates were 0.6% (65/11,400) in the Olympic Games and 0.6% (30/4,800) in the Paralympic Games, respectively. Considering both the Olympic and Paralympic Games, most cases (92/95; 97%) of athletes and team officials were observed in overseas travellers, whereas most cases among other GPIPCs were domestic residents (494/506; 98%), including those in specific occupations or roles such as security staff and torch relay participants (Table 3).

**Table 3:** Characteristics of reported SARS-CoV-2 infections related to the Tokyo 2020 Olympic and Paralympic Games, Japan, 1 July−19 September 2021

Characteristics		All (n = 804)		Athletes and team officials (n = 95)		Games’ organisers and media (n = 176)		Other GPIPCs (n = 506)		Unclassifiable personnel (n = 27)	
Variable		Median (IQR)		Median (IQR)		Median (IQR)		Median (IQR)		Median (IQR)	
Age in years		35 (27–45)		35 (26–45)		39 (32–49)		34 (25–44)		33 (25–49)	
Variable		Number	%	Number	%	Number	%	Number	%	Number	%
Sex	Female	194	24	30	32	27	15	132	26	5	19
	Male	610	76	65	68	149	85	374	74	22	81
Residence status	Overseas	232	29	92	97	128	73	12	2	0	0
	Domestic	572	71	3	3	48	27	494	98	27	100
Games’ type^a^	Olympic	NA		65	68	NA		NA		NA	
	Paralympic	NA		30	32	NA		NA		NA	

IQR: interquartile range; n: total number; NA: not applicable.

^a^ Data were not available for individuals other than Athletes and team officials.

The epidemic curve stratified by categories of Tokyo 2020 GPIPCs demonstrated a distribution peaking on 5 August, based on both date of symptom onset and date of diagnosis. The number of cases among athletes and team officials, most of whom were overseas travellers, began to increase on 14 July and peaked on 22–23 July based on date of diagnosis, which occurred 4 to 5 days after the peak arrivals for the Olympic Games, and peaked again on 21 August, 2 days after the peak arrivals for the Paralympic Games [5]. In contrast, the number of cases among other GPIPCs, most of whom were domestic residents, began to rise on 13 July, coinciding with the onset of the national epidemic in Japan [1], and peaked on 5 August based on date of diagnosis (Figure 1).

**Figure 1:**
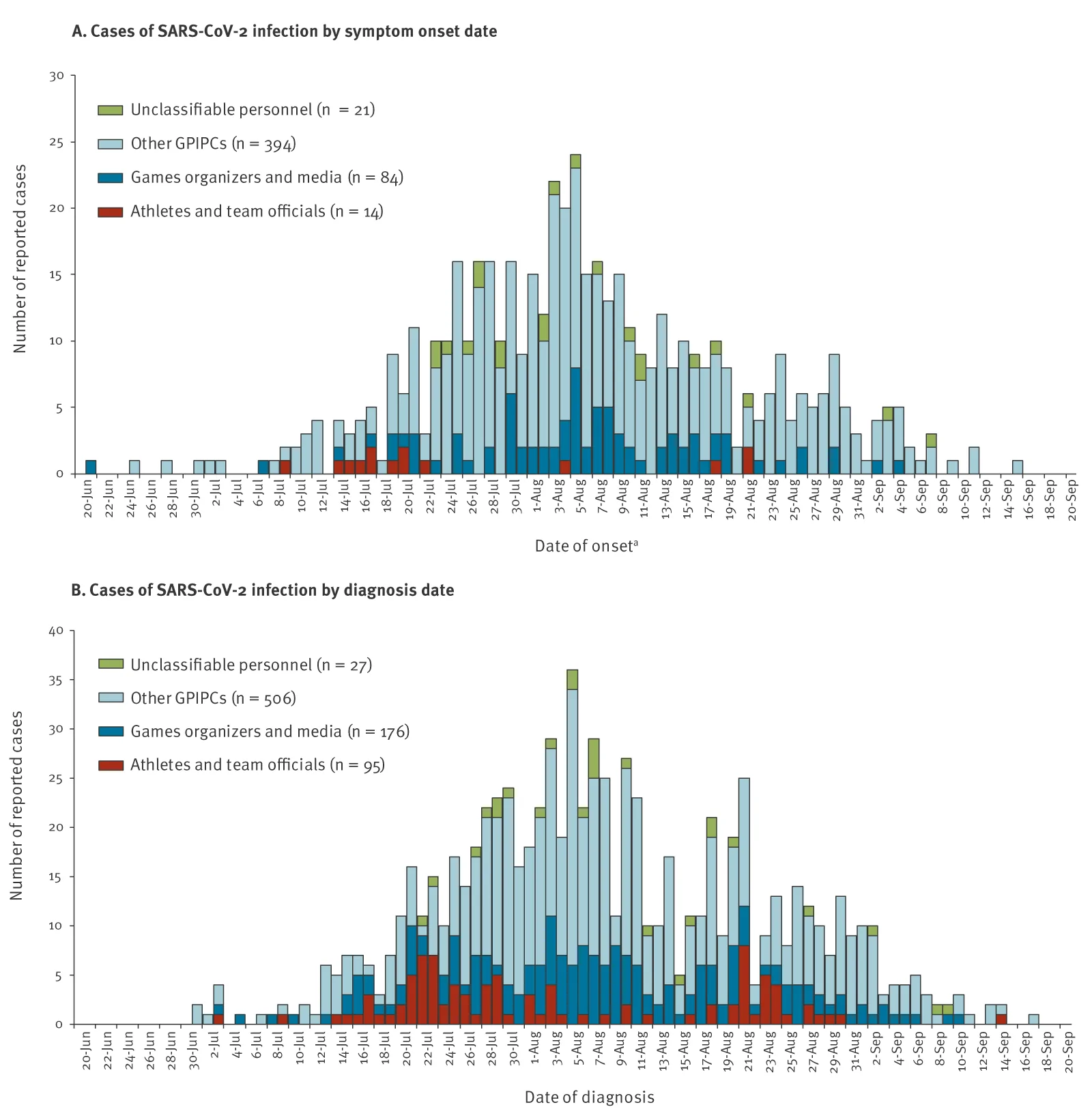
Reported cases of SARS-CoV-2 infection related to the Tokyo 2020 Olympic and Paralympic Games by GPIPCs’ category and dates of (A) symptom onset and (B) diagnosis, Japan, 1 July−19 September 2021 (n = 804)

GPIPCs: Games participants and in-person contributors.

^a^ Cases asymptomatic at the time of diagnosis are excluded.

Of 95 athlete and team officials’ cases, 92 had arrived in Japan from overseas. Among these 92 travellers, 92% (n = 85) tested positive on arrival at the airport or within 14 days after entry, and 87% (n = 80) were asymptomatic. In contrast, among the 176 Games’ organisers and media cases, only 35% (n = 45) of the 128, who had come from overseas were detected during this period; the remainder were diagnosed ≥ 15 days after entry (Table 4). Moreover, there appeared to be a larger proportion of individuals (48%) in this group detected when symptoms had already developed.

**Table 4:** Clinical characteristics and WGS status of reported SARS-CoV-2 infections related to Tokyo 2020, by case category and residence status, Japan, 1 July−19 September 2021 (n = 804)

Characteristics	Athletes and team officials			Games’ organisers and media			Other GPIPCs			Unclassifiable personnel	
	Overseas (n = 92)		Domestic (n = 3)	Overseas (n = 128)		Domestic (n = 48)	Overseas (n = 12)	Domestic (n = 494)		Overseas (n = 0)	Domestic (n = 27)
	Number	%^a^	Proportion^a^	Number	%^a^	Proportion^a^	Proportion^a^	Number	%^a^	Proportion^a^	Proportion^a^
**Symptoms**											
Symptomatic	12	13	2/3	60	47	24/48	6/12	388	79	0/0	21/27
Asymptomatic	80	87	1/3	68	53	24/48	6/12	106	21	0/0	6/27
**Timing of diagnosis^b^**											
Airport quarantine ≤ 14 days after entry	85	92	NA	45	35	NA	1/12	NA	NA	0/0	NA
≥ 15 days after entry	7	8	NA	83	65	NA	11/12	NA	NA	0/0	NA
**WGS status**											
Completed	75	82	0/3	103	80	28/48	5/12	91	18	0/0	2/27
Not performed or partially sequenced	17	18	3/3	25	20	20/48	7/12	403	82	0/0	25/27

GPIPC: Games participants and in-person contributor; NA: not applicable; n: total number; WGS: whole-genome sequencing.

^a^ The proportion of cases with each characteristic is provided in the form of a percentage or, when the denominator value is low, of a fraction.

^b^ Timing of diagnosis was applicable only to overseas cases, as entry quarantine applied only to overseas travellers.

### Molecular epidemiology

Among the 804 cases, whole-genome sequencing data were obtained for 304 (median age: 38 years, IQR: 29–47; 67 female/237 male). The proportions of cases with available genomic information in athletes and team officials, Games’ organisers and media, other GPIPCs and unclassifiable personnel were 79% (75/95), 74% (131/176), 19% (96/506) and two of 27, respectively (Table 4). Based on the phylogenetic analysis of the 304 cases, the majority (94%; 285/304) were infected with the Delta variant. The Alpha and Gamma variants accounted for only 5% (16/304) and 1% (3/304) of cases, respectively.

The Delta variant cases were further classified into two major groups: those belonging to the AY.29 lineage prevalent in Japan during the defined period, and those comprising other lineages. The AY.29 lineage was characterised by the presence of the mutations C5239T and T5514C, consistent with previous reports [14]. Data from the Global Initiative on Sharing Avian Influenza Data (GISAID) database indicated that Delta variant sequences harbouring both C5239T and T5514C mutations peaked in August 2021 and declined thereafter, with substantially lower levels observed after February 2022. Among 96,049 Delta variant sequences with both mutations registered in GISAID, 95,888 (99.83%) were reported from Japan, indicating that this genetic signature was predominantly observed in Japan. Hereafter, the AY.29 lineage, was considered as the domestic Delta variant, while other Delta lineages were considered as the overseas Delta variants in this study; 60% (182/304) of the sequenced strains belonged to the domestic Delta variant and 34% (103/304) to the overseas Delta variant.

The overseas Delta variant group mainly comprised SARS-CoV-2 positive samples from overseas visitors diagnosed at airport quarantine or within 14 days of entry. In this respect, the overseas Delta lineage dominated among the athletes and team officials (97%; 66/68) and Games’ organisers and media (29/34) GPIPCs diagnosed within 14 days of arrival (Figure 2, Table 5). On the other hand, as shown in the Supplementary Table, among Delta variant cases, the domestic Delta variant group predominantly comprised samples from residents of Japan (96%; 104/108). The domestic lineage was also prominent in overseas visitors diagnosed ≥ 15 days after entry (Figure 2), particularly among the Games’ organisers and media (97%; 63/65) (Figure 2, Table 5).

**Figure 2:**
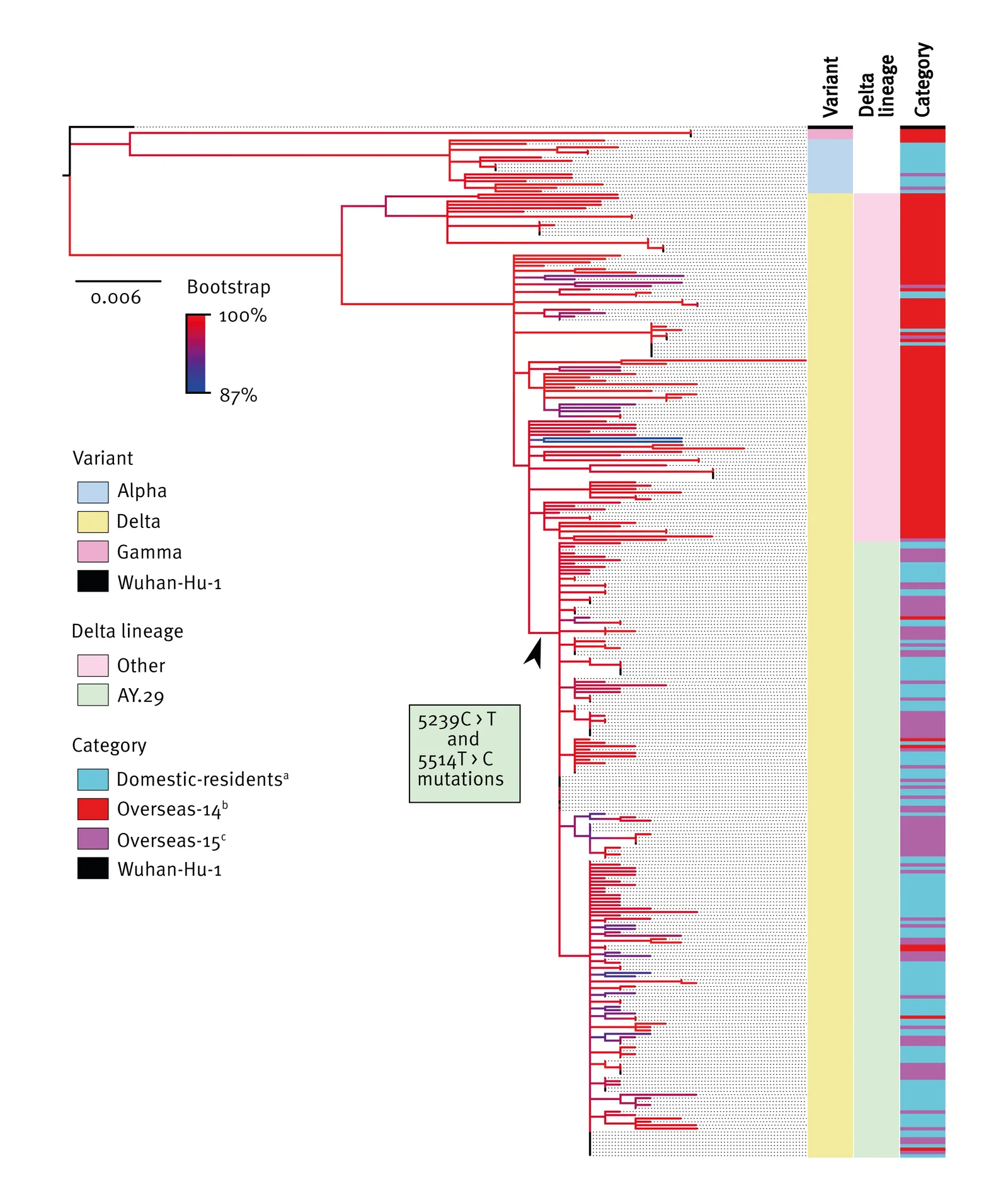
Phylogenetic analysis of isolates from reported cases of SARS-CoV-2 infection related to the Tokyo 2020 Olympic and Paralympic Games grouped by residence status and timing of diagnosis, Japan, 1 July−19 September 2021 (n = 304 case strains)

^a ^‘Domestic-residents’ refers to SARS-CoV-2 sequences from residents of Japan.

^b^ ‘Overseas-14d’ refers to SARS-CoV-2 infections in visitors from overseas, which were detected at airport quarantine or up to 14 days after entry in Japan.

^c ^‘Overseas-15d’ refers to SARS-CoV-2 infections in visitors from overseas, which were detected 15 days or more after entry in Japan.

A maximum-likelihood (ML) phylogenetic analysis was conducted on 304 whole-genome sequences of SARS-CoV-2 strains derived from infections related to Tokyo 2020, together with the Wuhan-Hu-1 reference strain sequence (accession number MN908947). In total, 935 single-nt variants (SNVs) were identified across 305 isolates.

**Table 5:** Distribution of domestic and overseas Delta variant strains according to residency status of cases and timing of entry of overseas cases, Japan, 1 July−19 September 2021 (n = 285)

Delta variants	Athletes and team officials		Games’ organisers and media		Other GPIPCs		Unclassifiable	
	Traveller (n = 73)	Resident (n = 0)	Traveller (n = 99)	Resident (n = 23)	Traveller (n = 5)	Resident (n = 83)	Traveller (n = 0)	Resident (n = 2)
Domestic (Delta AY.29 lineage)	6	0	68	21	4	81	0	2
Quarantine ≤ 14 days after entry	2	NA	5	NA	0	NA	0	NA
≥ 15 days after entry	4	NA	63	NA	4	NA	0	NA
Overseas (non-AY.29 lineage Delta strains)	67	0	31	2	1	2	0	0
Quarantine ≤ 14 days after entry	66	NA	29	NA	1	NA	0	NA
≥ 15 days after entry	1	NA	2	NA	0	NA	0	NA

GPIPC: Games participants and in-person contributor; NA: not applicable, as only overseas travellers were subjected to quarantine after entry in Japan.

Overseas athletes and team officials’ cases were typically diagnosed either during airport quarantine screening or within the mandated 14-day quarantine period, and the majority were infected with the overseas Delta variant (66/73; 90%). In contrast, among overseas Games’ organisers and media, more than half (63/99; 64%) were infected with the domestic Delta variant ≥ 15 days after entry (Table 5). As for the category of other GPIPCs, which included a large proportion of residents (83/88; 94%) infections caused by the Japan-dominant lineage of the Delta variant dominated (85/88; 97%).

Concerning overseas athletes, among the seven cases diagnosed ≥ 15 days after entry, five were whole genome sequenced. All five were infected with the Delta variant: four with the Japan-dominant Delta lineage and one with an overseas Delta lineage. The distribution of SARS-CoV-2 lineages in infections related to Tokyo 2020 by case category and residence status is shown in the Supplementary Table.

#### Clusters

In the overseas Delta variant group, three clusters comprising 5–11 cases were identified. One cluster included athletes and team officials, Games’ organisers and media and other GPIPCs, epidemiologically linked as members of the same national delegation or same sport, and comprising both overseas travellers and domestic residents. The remaining clusters comprised only overseas athletes and team officials epidemiologically linked as members of the same national delegation.

In the domestic Delta variant group, one distinct cluster of other GPIPCs was identified and consisted of security staff who were active in the same location and during the same period. A sensitivity analysis using a cluster threshold of ≥ 4 cases yielded similar findings (data not shown).

## Discussion

We examined the transmission dynamics of SARS-CoV-2 infections related to Tokyo 2020 by integrating epidemiological data with comparative genomic analysis of WGS data obtained through multiple surveillance systems, thereby enabling a comprehensive investigation. The results indicate that SARS-CoV-2 transmission into and within athletes and team officials was minimised by multiple infection prevention measures implemented immediately after their entry into Japan. This conclusion is supported first by the low attack rates among both Olympic and Paralympic athletes and team officials. Second, a considerable proportion of cases among athletes and team officials were detected either during airport quarantine screening or within the mandated 14-day quarantine period. Third, molecular epidemiology demonstrated limited infections caused by Japan-dominant lineage of SARS-CoV-2 among athletes and team officials. Moreover, instances of infection among individuals from different sports or countries were notably rare. This may have been caused by the highly unique infection-control conditions of the bubble system implemented for athletes, a highly controlled environment designed to minimise the risk of virus transmission by limiting interactions and subjecting individuals to active surveillance, including daily screening tests. These measures may also explain the lower proportion of symptomatic cases among athletes and team officials at the time of testing compared with other GPIPC categories, likely reflecting earlier detection through frequent screening tests.

In the category of other GPIPCs, which included a large proportion of infections caused by the Japan-dominant lineage of the Delta variant, we identified a cluster among security staff based on integrated epidemiological and genomic information. Potential opportunities for transmission, including work-related interactions, breaks, and accommodation among co-workers in closed settings, are key points at which infection-prevention and control measures should be strengthened to prevent outbreaks of human-to-human infectious diseases, which may directly or indirectly affect large-scale events. Furthermore, a notable subset of infections caused by Japan-dominant lineage of the Delta variant among overseas Games’ organisers and media was identified. These findings suggest that some Games’ organisers and media personnel arriving from abroad may have contracted their infections within Japan, likely reflecting differences in the intensity of infection control measures compared with athletes and team officials. In addition, there is a possibility that the measures set out in the Playbook and by relevant authorities were not adequately followed or were not sufficiently delivered to these groups. Relevant public health authorities and event organisers need to recognise such groups as risk populations, given their potential contribution to domestic outbreaks, international exportation of infectious diseases, and the emergence or amplification of new variants during international mass gatherings [15-17]. Targeted risk communication should therefore be delivered to these groups both before and during the event.

This report highlights the value of integrating epidemiological and genomic information to provide insights into the transmission of SARS-CoV-2. This initiative contributed to an intra- and after-action review of the effectiveness of public health interventions implemented during the Games. During Tokyo 2020, we monitored the importation of new SARS-CoV-2 lineages, including variants of concern, from abroad and their potential spread in Japan using genomic surveillance. Moreover, immediately following preliminary findings of the molecular analysis during the Olympic Games, we conducted a reassessment of the Games and implemented additional measures for the Paralympic Games [5]. Previous reports emphasised the importance of whole-genome-based network/phylogeny analysis to elucidate possible transmission routes during outbreaks in mass gathering settings, providing crucial information for preventing future outbreaks [9,18,19]. This report, showcasing the dynamics of infectious-disease transmission during international large-scale sports events through molecular epidemiology, is extremely valuable. However, challenges persist. Comprehensive genomic data collection incurs significant resources, including cost and substantial effort by GPIPCs, making it unfeasible in all settings. The strategic task ahead involves demonstrating efficient genomic surveillance with systematic case selection and effective genome information collection for outbreak-associated cases, together with the integration of epidemiological information within public health management. There is a need for strengthened preparedness and response capacities for infectious diseases at future international mass gatherings, especially in the context of public health crises such as the COVID-19 pandemic.

There were several limitations in this study. First, cases without genomic and epidemiological information created difficulties in understanding the association among cases. Concerning genomic data, this may have been due to Cq values over 32 in RT-PCR or to unsuccessful sequencing. Regarding this, many specimens had already been sequenced at commercial laboratories before being submitted to the NIID, so the overall sequencing success proportion could not be accurately determined. Lack of genomic data was more pronounced in the group of other GPIPCs, as especially athletes and team officials and groups with closer proximity to athletes were prioritised for sequencing and epidemiological investigation. Consequently, we might have underestimated potential transmission routes in this group. It should also be noted that attack rates could only be determined for athletes and team officials, as accurate denominator data were not available for the other case categories. Second, as this analysis relied solely on cases reported in the Tokyo Metropolitan area, the nationwide transmission dynamics related to Tokyo 2020 may not have been fully described. However, given the concentration of Tokyo 2020s main venues in the Tokyo Metropolitan area, and the fact that all cases under the jurisdiction of TOCOG were reported in Tokyo, this report provides an effective representation of the transmission dynamics among the core at-risk population associated with Tokyo 2020.

## Conclusion

Molecular epidemiology demonstrated limited SARS-CoV-2 transmission among GPIPCs associated with Tokyo 2020 in Japan, with layered infection prevention, early detection, rapid isolation, and targeted efforts for Olympic and Paralympic athletes. Combining epidemiological data with comparative genomic analysis provides a powerful tool for evaluating the effectiveness of risk-reduction strategies in large international mass gatherings.

## Data Availability

Of the sequences used in this analysis, 33 genomes were identified through routine SARS-CoV-2 genomic surveillance or genomic surveillance at quarantine stations. Subsequent epidemiological investigations identified the corresponding cases as stakeholders of the Tokyo 2020 Olympic and Paralympic Games. These genomes have been deposited in the Global Initiative on Sharing All Influenza Data (GISAID) database. The corresponding accession numbers are provided in Supplementary Table S2. The remaining sequences, which were obtained through surveillance specifically conducted among stakeholders of the Tokyo 2020 Olympic and Paralympic Games, are not publicly available because of privacy considerations, including the potential for individual cases to be identified when genomic data are combined with epidemiological information.

## Supplementary Data

192000 Supplement S1

## Supplementary Data

15000 Table S2
