# Supplementary material for: Minimised SARS-CoV-2 transmission during Tokyo 2020 Olympic and Paralympic Games: insights from enhanced integrated surveillance, Japan, July to September 2021
Source: Euro Surveill. 2026 Oct 22;31(42):2600009. doi: 10.2807/1560-7917.ES.2026.31.42.2600009 (PMC13695007; doi:10.2807/1560-7917.ES.2026.31.42.2600009)
Supplement: Supplementary file 1 [file 26-00009_SAITO_SupplementaryTable.pdf]

Supplementary Table. Characteristics and proportion of SARS-CoV-2 lineages in reported SARS-CoV-2 infections related to Tokyo 2020 with available completed whole genome sequences, by case category and residence status (n=304)

This supplementary material is hosted by *Eurosurveillance* as supporting information alongside the article "Minimised SARS-CoV-2 Transmission during Tokyo 2020 Olympic and Paralympic Games: Insights from Enhanced Integrated Surveillance, Japan, July to September 2021", on behalf of the authors, who remain responsible for the accuracy and appropriateness of the content. The same standards for ethics, copyright, attributions and permissions as for the article apply. Supplements are not edited by *Eurosurveillance* and the journal is not responsible for the maintenance of any links or email addresses provided therein.

	Athletes and team officials				Games organizers and media				Other stakeholders				Unclassifiable personnel			
	Overseas (n=75)		Domestic (n=0)		Overseas (n=103)		Domestic (n=28)		Overseas (n=5)		Domestic (n=91)		Overseas (n=0)		Domestic (n=2)	
**Age (y)**																
**Median (IQR)**	34	26-44	NA	NA	39	32-47	46.5	31-51	35	32-35	38	28-49	NA	NA	21	18-24
	n	%	n	%	n	%	n	%	n	%	n	%	n	%	n	%
**Sex**																
Female	23	31	0	0	14	14	3	11	0	0	26	29	0	0	1	50
Male	52	69	0	0	89	86	25	89	5	100	65	71	0	0	1	50
**Timing of diagnosis***																
Airport quarantine or within 14 days after entry	70	93	NA	NA	36	35	NA	NA	1	20	NA	NA	0	0	NA	NA
≥15 days after entry	5	7	NA	NA	67	65	NA	NA	4	80	NA	NA	0	0	NA	NA
**Variant lineage**																

Domestic[a]	6	8	0	0	68	66	21	75	4	80	81	89	0	0	2	100
Overseas[b]	67	89	0	0	31	30	2	7	1	20	2	2	0	0	0	0
Alpha	1	1	0	0	2	2	5	18	0	0	8	9	0	0	0	0
Gamma	1	1	0	0	2	2	0	0	0	0	0	0	0	0	0	0

NA, not applicable.

* Timing of diagnosis was applicable only to overseas cases, as entry quarantine applied only to overseas travellers.

a Domestic Delta variant lineage, defined as the Delta variant lineage, those belonging to the AY.29 lineage, and prevalent in Japan during the defined period.

b Overseas Delta variant lineage, defined as including all other lineages.
